# Supplementary material for: Tandem LC-MS Identification of Antitubercular Compounds in Zones of Growth Inhibition Produced by South African Filamentous Actinobacteria
Source: Molecules. 2023 May 23;28(11):4276. doi: 10.3390/molecules28114276 (PMC10254512; doi:10.3390/molecules28114276)
Supplement: Supplementary file 1 [file molecules-28-04276-s001.zip › molecules-2278924-supplementary.pdf]

Table S1: Antimycobacterial activity against *M. aurum* A+ from agar overlay assays of selected filamentous actinobacteria grown in six different growth media.

+ = zone of growth inhibition observed   - = no zone of inhibition observed

Test Strain	Middlebrook	Czapek	DSMZ #553	Modified Bennett's	ISP-2	JCM #61
Actinomadura napierensis B60^{T}	+	-	-	-	+	-
Actinomadura rudentiformis HMC1^{T}	-	-	-	-	-	-
Actinomadura strain M2	-	+	-	-	-	
Actinomadura strain M27	-	-	+	-	-	-
Amycolatopsis circi S1.3^{T}	-	-	+	+	+	-
Amycolatopsis strain CP1B7Th	+	+	+	+	+	+
Amycolatopsis strain 3B4Tu	+	+	+	+	+	+
Kribbella speibonae SK5	-	-	-	+	+	-
Kribbella strain 1B8Tu	-	-	+	+	+	-
Microbispora strain SMA-HA3	-	-	-	-	-	-
Micromonospora equina Y22^{T}	-	-	-	-	-	-
Micromonospora strain Muiz4Y	-	-	+	-	-	-
Micromonospora strain M27	-	-	+	-	+	-
Micromonospora tulbaghiae TVU1^{T}	-	-	-	-	-	-
Nocardia strain 2017S B10 Fri	-	-	+	-	-	-
Nocardia strain 2017S B3Th	-	-	-	-	-	-
Nocardia strain N12	-	-	+	-	-	-
Nocardia strain N15	-	-	-	-	-	-
Nocardia rhamnosiphila GM202^{T}	-	-	-	-	-	-

Nocardia strain N1	-	-	-	-	-	-
Nonomuraea strain QMC26	+	+	-	-	-	-
Nonomuraea strain SMA-BO4	-	-	-	-	-	-
Streptomyces africanus CPJVP-H^{T}	-	-	-	-	+	-
Streptomyces strain 268	-	+	-	-	-	-
Streptomyces strain JBGM7	-	-	-	+	-	-
Streptomyces fractus MV32^{T}	-	-	+	-	-	-
Streptomyces strain #15	-	-	+	+	-	-
Streptomyces strain 1J1	-	-	+	-	-	-
Streptomyces strain B1	+	+	+	+	+	-
Streptomyces strain B2	+	+	+	+	+	-
Streptomyces strain C23	-	-	+	-	+	-
Streptomyces strain C3	-	-	-	-	+	-
Streptomyces strain CW2	-	-	+	+	+	+
Streptomyces strain CW5	-	-	-			+
Streptomyces strain HMC5	-	-	-	-	-	+
Streptomyces strain M8	-	-	-	-	-	-
Streptomyces strain PR10	-	+	+	+	+	-
Streptomyces strain PR3	+	+	+	+	+	+
Streptomyces strain PR36	-	-	+	+	-	-
Streptomyces strain PR4	-	-	+	+	+	+
Streptomyces strain Y10	-	-	+	-	+	+
Streptomyces strain Y12	-	-	+	+	-	+
Streptomyces strain Y30	-	-	+	+	-	+

Table S2. Accurate masses of identified antibiotics in the zones of inhibition and liquid culture crude extracts of active strains with theoretical monoisotopic masses, accurate mass differences and mass error.

Liquid: Small scale liquid cultures (25 mL).

Agar: agar-plate cultivation

* The cosine score is generated by GNPS molecular networking and is a measure of spectral similarity, with 1 being an identical match between the query spectrum and the GNPS library spectrum.

Strain name	Growth medium	Source	Compound	Cosine score*	Theoretical monoisotopic mass (Da)	Experimental Mass (Da)	Accurate mass difference (Da)	Mass Error (ppm)
Kribbella strain 1B8Tu	DSMZ #553	Agar	Actinomycin D	0.78	1254.6284	1254.6314	-0.0030	-2.391
		Liquid		0.78	1254.6284	1254.6310	-0.0026	-2.072
Streptomyces strain C23	ISP-2	Agar	Puromycin	0.96	471.2230	471.2236	-0.0006	-1.273
		Liquid		0,98	471.2230	471.2231	-0.0001	-0.212
Streptomyces strain CW2	ISP-2	Agar	Actinomycin D	0.84	1254.6284	1254.6284	0.0000	0.000
		Liquid		0.8	1254.6284	1254.6310	-0.0026	-2.072
	JCM #61	Agar		0.84	1254.6284	1254.6298	-0.0014	-1.116
		Liquid		0.79	1254.6284	1254.6294	-0.0010	-0.797
Streptomyces strain PR3	ISP-2	Agar	Valinomycin	0.9	1110.6311	1110.6348	-0.0037	-3.331
		Liquid		0.95	1110.6311	1110.6331	-0.0020	-1.801
	DSMZ #553	Agar		0.94	1110.6311	1110.6315	-0.0004	-0.360
		Liquid		0.88	1110.6311	1110.6300	0.0011	0.990
	Modified Bennett's	Agar		0.79	1110.6311	1110.6302	0.0009	0.810
		Liquid		0.92	1110.6311	1110.6346	-0.0035	-3.151
	Czapek	Agar		0.92	1110.6311	1110.6348	-0.0037	-3.331
		Liquid		0.88	1110.6311	1110.6348	-0.0037	-3.331
	JCM #61	Agar		0.8	1110.6311	1110.6331	-0.0020	-1.801
		Liquid		0.72	1110.6311	1110.6315	-0.0004	-0.360
	Middlebrook	Agar		0.82	1110.6311	1110.6300	0.0011	0.990
		Liquid		0.81	1110.6311	1110.6302	0.0009	0.810
Streptomyces strain PR10	ISP-2	Agar	Actinomycin D	0.77	1254.6284	1254.6262	0.0022	1.754
		Liquid		0.78	1254.6284	1254.6294	-0.0010	-0.797
	DSMZ #553	Agar		0.86	1254.6284	1254.626	0.0024	1.913
		Liquid		0.78	1254.6284	1254.6289	-0.0005	-0.399
	Modified Bennett's	Agar		0.87	1254.6284	1254.6283	0.0001	0.080
		Liquid		0.8	1254.6284	1254.6298	-0.0014	-1.116
	Czapek	Agar		0.84	1254.6284	1254.6300	-0.0016	-1.275
		Liquid		0.72	1254.6284	1254.6301	-0.0017	-1.355
Streptomyces strain PR36	DSMZ #553	Agar	Valinomycin	0.8	1110.6311	1110.6346	-0.0035	-3.151
		Liquid		0.85	1110.6311	1110.629	0.0021	1.891

Table S3: *In vitro* antitubercular activity (nM) against *M. tuberculosis* H37Rv^T of selected South African actinobacterial strains. Two technical repeats, one biological repeat.

Strain Name	Growth Medium	7H9_ADC_GLU_TX	7H9_CAS_GLU_TX	7H9_ADC_GLU_TW
Actinomadura napierensis B60^T	Middlebrook	> 62.5	26.1	> 62.5
Actinomadura napierensis B60^T	ISP-2	2.3	1.3	1.6
Actinomadura strain M2	Czapek	> 62.5	6.9	25.7
Actinomadura strain M27	DSMZ #553	> 62.5	21.7	> 62.5
Amycolatopsis circi S1.3^T	Modified Bennett's	> 62.5	> 62.5	5.2
Amycolatopsis circi S1.3^T	DSMZ #553	> 62.5	30.4	30.9
Amycolatopsis circi S1.3^T	ISP-2	> 62.5	6.8	50.2
Amycolatopsis strain CP1B7Th	ISP-2	> 62.5	> 62.5	> 62.5
Amycolatopsis strain CP1B7Th	DSMZ #553	> 62.5	> 62.5	> 62.5
Amycolatopsis strain CP1B7Th	Modified Bennett's	> 62.5	> 62.5	> 62.5
Amycolatopsis strain CP1B7Th	Czapek	> 62.5	> 62.5	> 62.5
Amycolatopsis strain CP1B7Th	JCM #61	> 62.5	> 62.5	> 62.5
Amycolatopsis strain CP1B7Th	Middlebrook	> 62.5	> 62.5	> 62.5
Amycolatopsis strain 3B4Tu	ISP-2	> 62.5	> 62.5	> 62.5
Amycolatopsis strain 3B4Tu	DSMZ #553	> 62.5	> 62.5	23.7
Amycolatopsis strain 3B4Tu	Modified Bennett's	54.4	29.9	0.79

Amycolatopsis strain 3B4Tu	Czapek	> 62.5	> 62.5	> 62.5
Amycolatopsis strain 3B4Tu	JCM #61	> 62.5	> 62.5	> 62.5
Amycolatopsis strain 3B4Tu	Middlebrook	> 62.5	> 62.5	> 62.5
Kribbella speibonae SK5	Modified Bennett's	> 62.5	26.6	> 62.5
Kribbella speibonae SK5	ISP-2	> 62.5	9.8	> 62.5
Kribbella strain 1B8Tu	ISP-2	> 62.5	> 62.5	> 62.5
Kribbella strain 1B8Tu	Modified Bennett's	> 62.5	> 62.5	15.6
Micromonospora strain Muiz4Y	DSMZ #553	> 62.5	> 62.5	> 62.5
Micromonospora strain 1J1	DSMZ #553	> 62.5	5.9	> 62.5
Micromonospora strain M27	DSMZ #553	> 62.5	> 62.5	> 62.5
Micromonospora strain M27	ISP-2	> 62.5	> 62.5	> 62.5
Nocardia strain B10S Fri	DSMZ #553	61.1	40.5	52.9
Nocardia strain N12	DSMZ #553	> 62.5	> 62.5	> 62.5
Nonomuraea strain QMC26	Middlebrook	> 62.5	31.1	> 62.5
Nonomuraea strain QMC26	Czapek	> 62.5	31.5	> 62.5
Streptomyces africanus CPJVR-H^{T}	ISP-2	3.4	> 62.5	5.2
Streptomyces strain #15	DSMZ #553	> 62.5	24.0	> 62.5
Streptomyces strain #15	Modified Bennett's	> 62.5	> 62.5	> 62.5
Streptomyces strain 268	Czapek	> 62.5	> 62.5	> 62.5
Streptomyces strain B1	Czapek	> 62.5	31.0	> 62.5

Streptomyces strain B1	Modified Bennett's	> 62.5	6.0	27.8
Streptomyces strain B1	ISP-2	> 62.5	9.6	43.1
Streptomyces strain B1	Middlebrook	> 62.5	> 62.5	> 62.5
Streptomyces strain B1	DSMZ #553	> 62.5	> 62.5	> 62.5
Streptomyces strain B2	Middlebrook	> 62.5	> 62.5	> 62.5
Streptomyces strain B2	DSMZ #553	> 62.5	> 62.5	> 62.5
Streptomyces strain B2	Modified Bennett's	> 62.5	29.7	23.2
Streptomyces strain B2	Czapek	> 62.5	> 62.5	> 62.5
Streptomyces strain B2	ISP-2	> 62.5	29.5	> 62.5
Streptomyces strain C3	ISP-2	> 62.5	29.5	> 62.5
Streptomyces strain CW5	JCM #61	0.6	0.2	< 0.12
Streptomyces strain HMC5	JCM #61	25.6	2.8	7.9
Streptomyces strain JBGM7	Modified Bennett's	> 62.5	26.5	> 62.5
Streptomyces strain M8	DSMZ #553	> 62.5	6.1	6.5
Streptomyces fractus MV32^T	DSMZ #553	0.8	< 0.12	< 0.12
Streptomyces strain PR4	DSMZ #553	> 62.5	14.9	> 62.5
Streptomyces strain PR4	Modified Bennett's	2.1	2.4	0.397
Streptomyces strain PR4	ISP-2	> 62.5	> 62.5	> 62.5
Streptomyces strain PR4	JCM #61	> 62.5	> 62.5	> 62.5
Streptomyces strain Y10	DSMZ #553	< 0.12	< 0.12	< 0.12
Streptomyces strain Y10	JCM #61	0.5	< 0.12	< 0.12
Streptomyces strain Y10	ISP-2	> 62.5	> 62.5	> 62.5
Streptomyces strain Y12	Modified Bennett's	> 62.5	2.3	> 62.5
Streptomyces strain Y12	JCM #61	> 62.5	16.2	> 62.5
Streptomyces strain Y12	DSMZ #553	> 62.5	> 62.5	> 62.5

Streptomyces strain Y30	Modified Bennett's	> 62.5	16.5	> 62.5
Streptomyces strain Y30	DSMZ #553	> 62.5	> 62.5	> 62.5

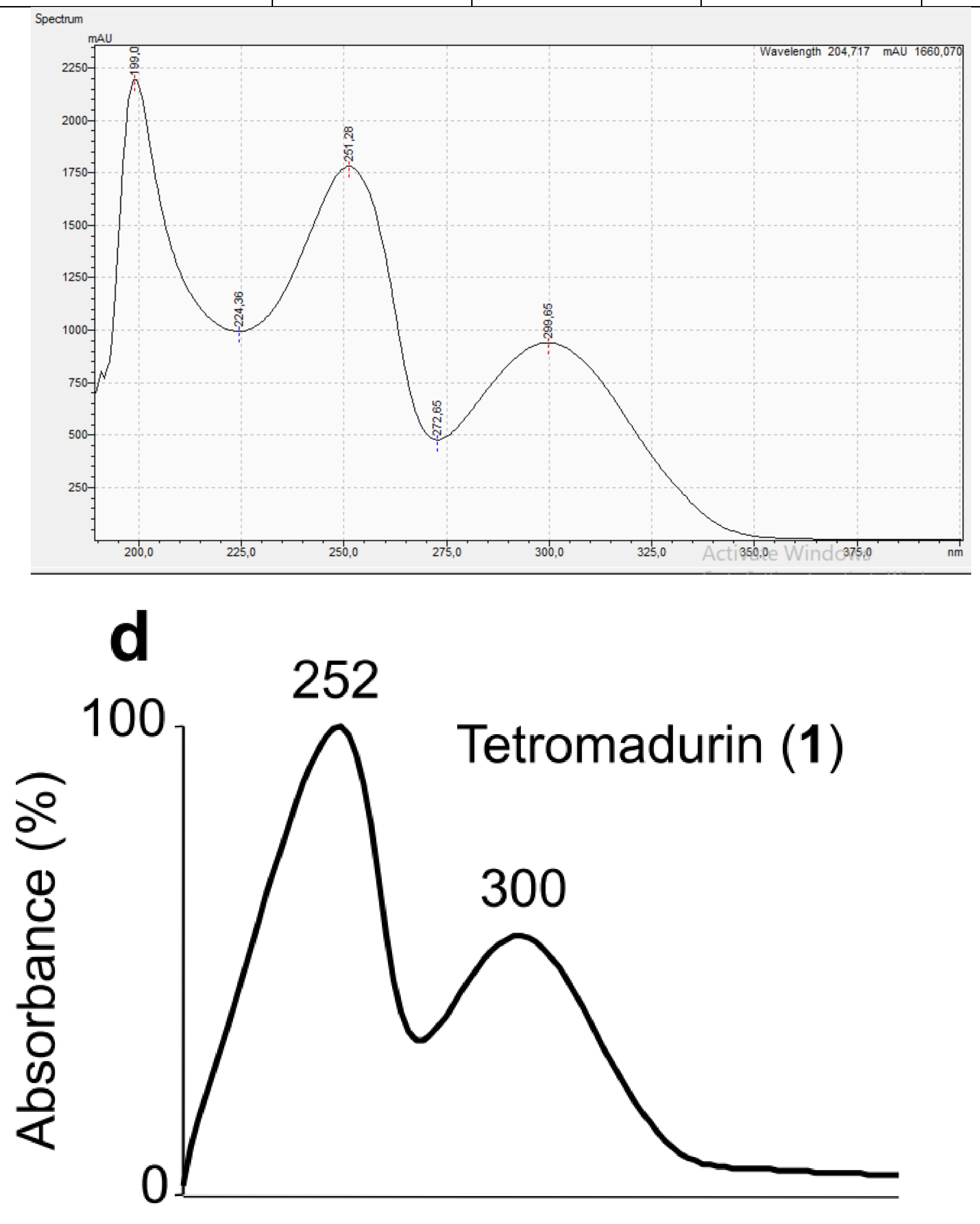

Figure S1:Comparison of experimentally obtained UV spectrum of identified tetromadurin (top) vs published spectrum of tetromadurin (bottom) showing a strong match between the two [28].

Table S4: Carbon and hydrogen NMR spectroscopic data of tetromadurin presented in original isolation paper by [29] compared to the experimental data measured from tetromadurin obtained in this study.

Carbon #	δC (100 MHz, CDCl3) [29]	Type	δH (400 MHz, CDCl3) [29]	δC (100 MHz, CDCl3) (Experimental)	δH (400 MHz, CDCl3) (Experimental
C-1	171.2	C	-	-	-
C-2	96.2	C	-	-	-
C-3	196.0	C	-	-	-
C-4	83.3	CH	4.67 br s	83.3	4.68
C-5	37.2	CH	2.45, t, 9.8	36.96	2.46
C-6	43.5	CH	1.64	43.3	1.66
C-7	76.3	CH	4.02, br s	76.4	4.04
C-8	35.9	CH	1.52, ??	35.7	1.53
C-9	34.9	CH2	0.98<br>1.36	34.79	0.99<br>1.36
C-10	36.1	CH	2.72, dq, 3.6, 9.8	35.79	2.74
C-11	137.6	CH	5.24, d, 10.4	137.4	5.23
C-12	135.9	C	-	-	-
C-13	85.0	CH	3.62-2.72	84.8	3.69
C-14	32.3	CH2	1.18<br>1.80	32.18	1.22<br>1.80
C-15	31.3	CH2	1.20<br>1.34	31.1	1.24<br>1.42
C-16	31.2	CH2	1.58	31.0	1.5
C-17	92.1	CH	3.42, d, 10.8	92.0	3.44
C-18	134.5	C	2.26, m		
C-19	131.1	CH	5.84, br dd, 6.0, 8.0	131.2	5.85
C-20	32.2	CH2	2.26, m	32.1	2.27
C-21	83.3	CH	3.62-3.72	83.3	3.66
C-22	40.2	CH	1.80	40.4	1.84
C-23	44.5	CH2	1.30<br>1.96, dd, 9.4, 12.4		
C-24	82.4	C	-	-	-
C-25	82.9	CH	3.62 - 3.72	-	-
C-26	25.2	CH2	1.85 – 1.95	25.96	1.81
C-27	22.8	CH2	1.66<br>1.80	22.49	1.66<br>1.83
C-28	81.3	CH	3.62-3.72	81.2	3.73
C-29	70.5	CH	3.97, dt, 2.6, 7.2		
C-30	28.2	CH2	1.23<br>1.35	27.9	1.42
C-31	10.5	CH3	0.93, t, 7.2	10.2	0.95
C-32	25.6	CH3	1.06, s	25.37	1.08
C-33	16.2	CH3	1.00, d, 6.4	16.07	1.02
C-34	10.4	CH3	1.72, s	10.2	1.74
C-35	17.3	CH3	0.66, d, 7.6	17.1	0.66
C-36	55.9	CH2	3.94,<br>4.25, d, 11.2	55.6	3.94<br>4.27
C-37	17.8	CH3	0.91, d, 7.2	17.56	0.93
C-38	65.6	CH2	3.88<br>4.36, dd, 2.6, 13.0	65.4	3.88<br>4.38

C-39	180.2	C	-	-	-
C-40	153.5	C	-	-	-
C-41	90.1	CH2	4.78,<br>5.18, d, 2.8	90.1	4.80<br>5.20
C-42	58.9	CH3	3.34	58.5	3.35

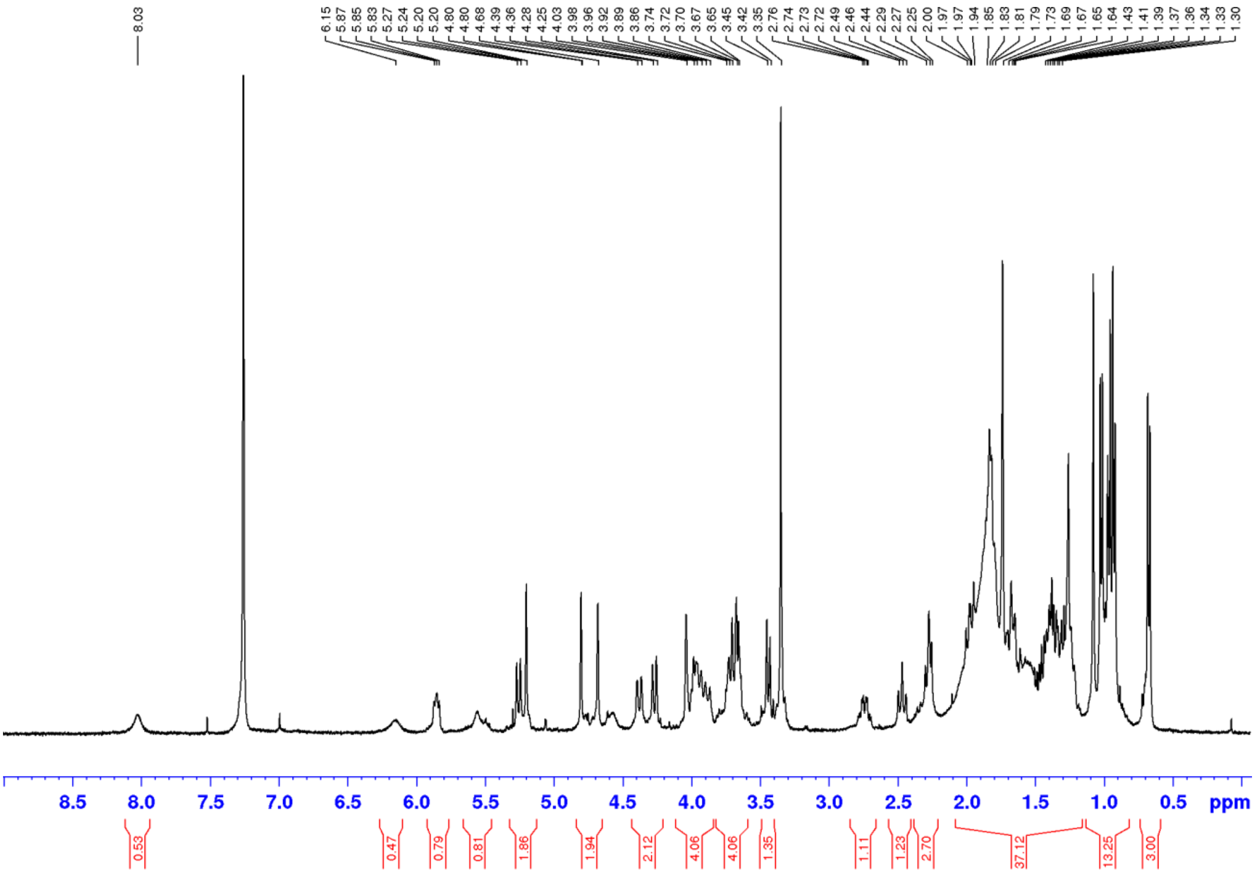

Figure S2. ^{1}H NMR spectrum of tetromadurin (400 MHz, CDCl3)

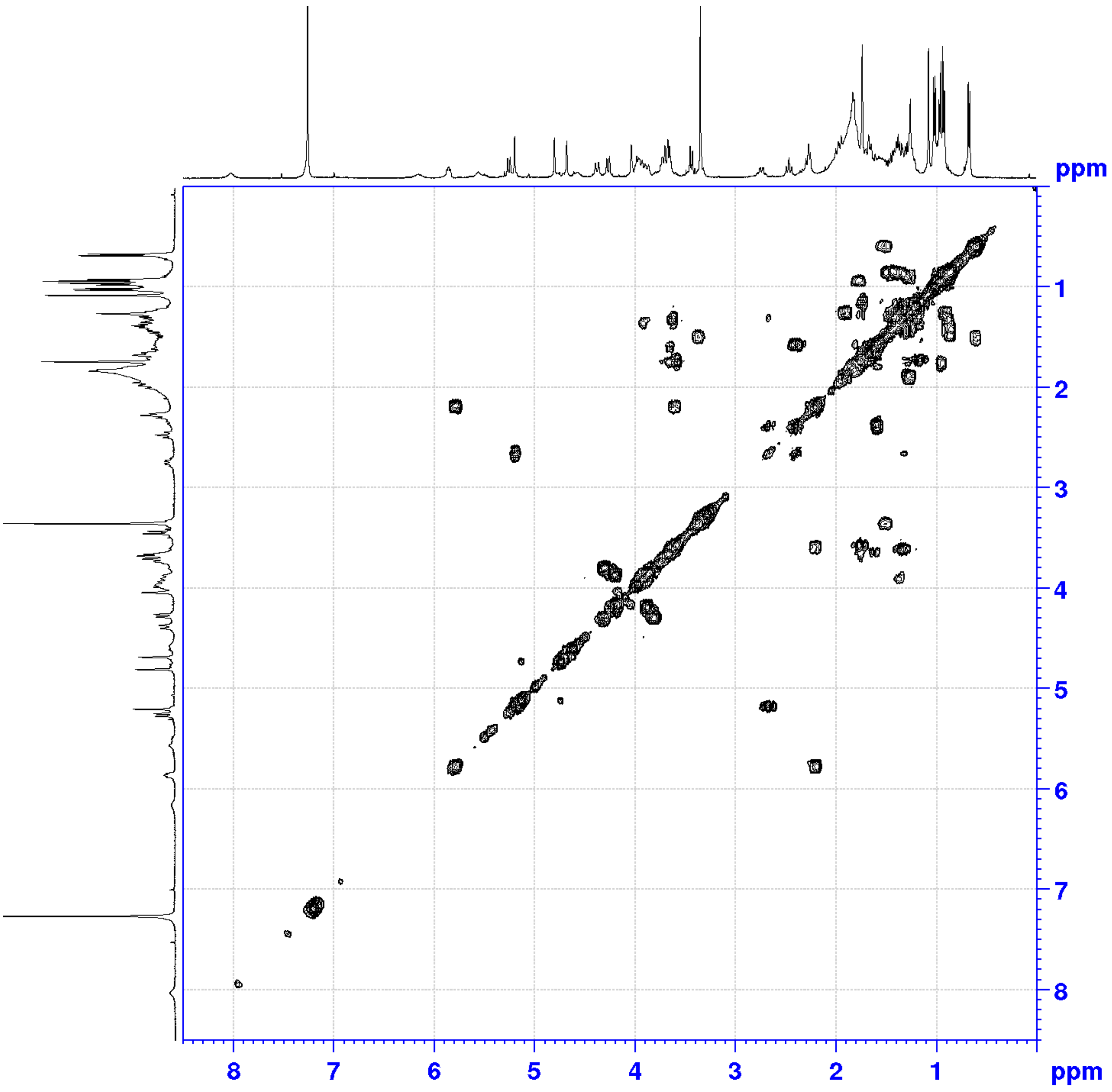

Figure S3. ^{1}H-^{1}H COSY spectrum of tetromadurin

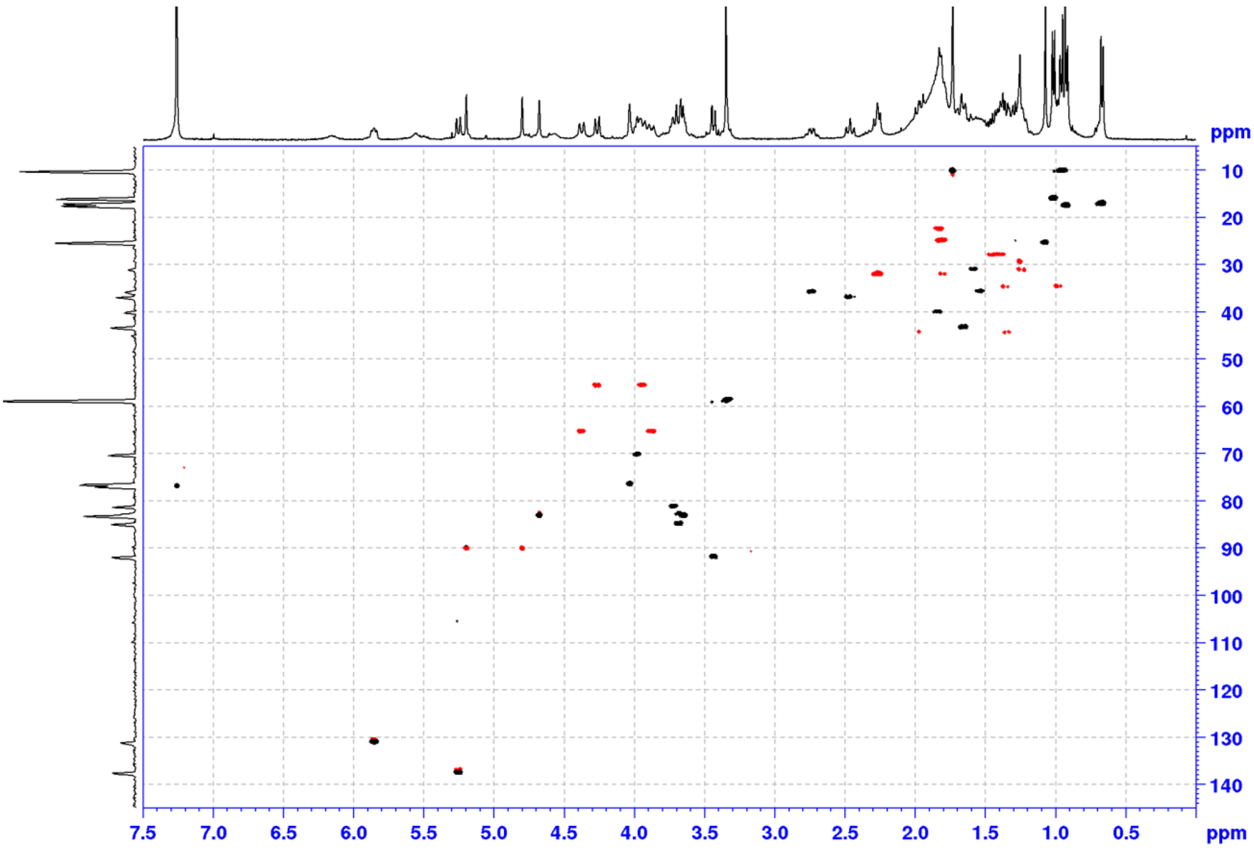

Figure S4. HSQC NMR spectrum of tetromadurin

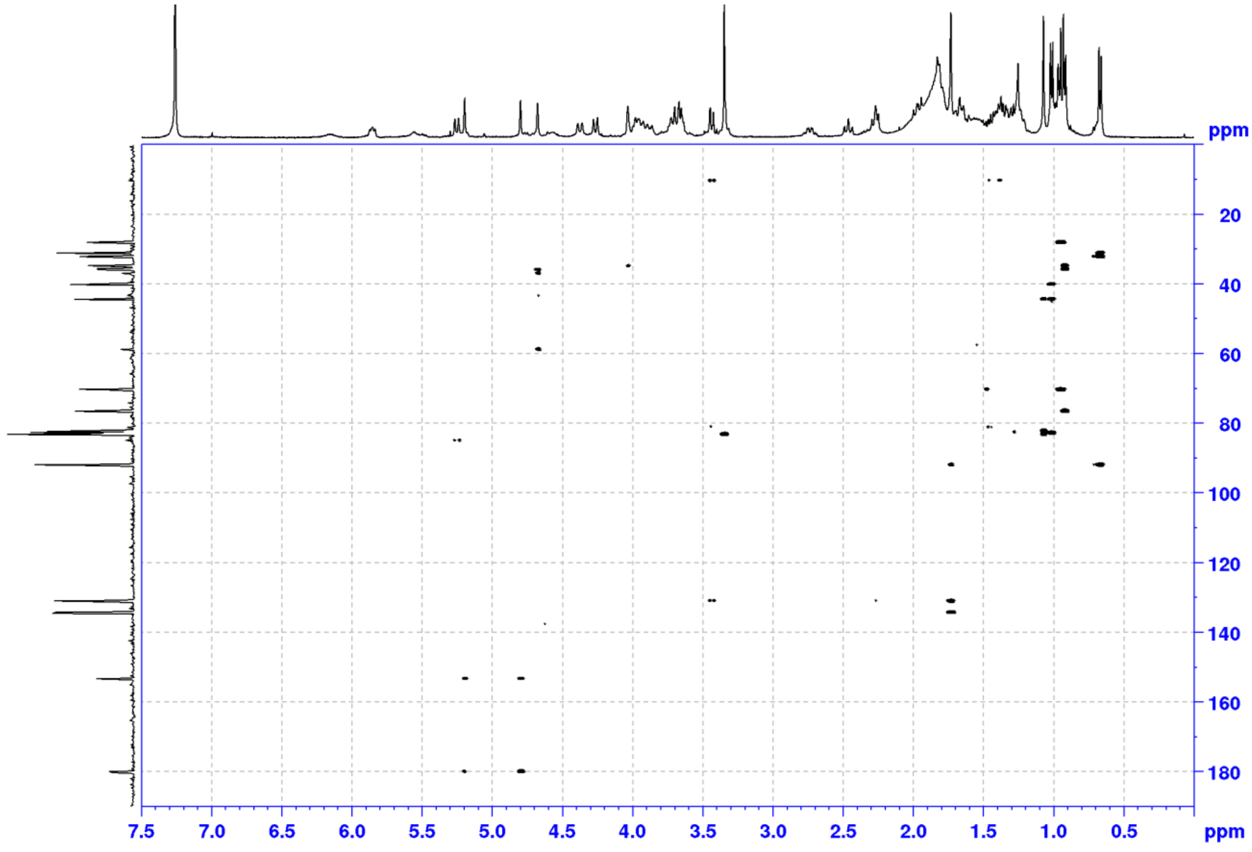

Figure S5. HMBC NMR spectrum of tetromadurin
